# Reply to the Letter to the Editor: GPT-4o in radiology—a review of label extraction accuracy and clinical applications in upper extremity imaging

**DOI:** 10.1007/s00330-026-12350-9

**Published:** 2026-02-04

**Authors:** Hanna Kreutzer, Sven Nebelung

**Affiliations:** 1 Department of Diagnostic and Interventional Radiology, University Hospital Aachen, Aachen, Germany; 2 Lab for Artificial Intelligence in Medicine, Department of Diagnostic and Interventional Radiology, University Hospital Aachen, Aachen, Germany

Dear Editor,

We thank Drs. Zhang and Zhang for their thoughtful commentary on our article “Large language model-based uncertainty-adjusted label extraction for artificial intelligence model development in upper extremity radiography.” We appreciate the opportunity to clarify several aspects of our work and to further elaborate on its methodological and translational implications.

First, regarding scope and generalizability, we agree that extending our approach to additional regions and conditions represents an important next step. In contrast to more widely researched areas, such as chest radiography [1, 2], our study deliberately focused on sparsely investigated upper-extremity anatomy, where multi-label datasets are particularly scarce and manual labeling is resource-intensive. As each region requires dedicated template design, quality assurance, and model training, a comprehensive multi-region expansion was beyond the scope of this proof-of-concept. Nonetheless, the proposed pipeline is intentionally modular, and we concur that broader, multi-institutional datasets will be essential to further strengthen generalizability.

Second, we appreciate the suggestion to adopt more granular categories of diagnostic uncertainty. In principle, differentiating “probable,” “possible,” or “unlikely” is appealing. In practice, however, uncertain statements were rare in both internal and external datasets, with only small numbers identified across several thousand reports. Subdividing uncertainty into multiple classes would therefore have yielded insufficient statistical power. Our binary uncertainty framework should thus be viewed as a methodological baseline that future large-scale collaborations may refine once larger, higher-prevalence datasets become available.

Third, with respect to the size and validation of the test sets and potential bias, we share the correspondents’ view that the way reference labels are established is crucial. By design, our internal dataset was split into 64%/16%/20% training/validation/test [3]. Thus, the test sets represent a subset of 20% of all cases. However, in absolute terms, the internal test sets still comprised *n* = 233 clavicle, *n* = 745 elbow, and *n* = 393 thumb examinations, complemented by external test sets of 300 cases per region, which we consider sufficiently large to derive stable and statistically meaningful performance estimates [4]. All internal and external test-set labels were manually checked against the original radiologic reports. Notably, this task required verifying correspondence between textual statements and structured labels, not interpreting or re-reading the radiographs themselves. The risk of inter-reader variability is therefore considerably lower than in segmentation or grading tasks [5]. Nevertheless, we agree that future work could be strengthened through multi-reader verification and formal agreement analysis.

Fourth, regarding clinical integration, we share the authors’ interest in how such systems can be used in real-world workflows. Although our primary goal was methodological, i.e., demonstrating that large language models (LLMs) can generate accurate, uncertainty-aware labels that enable competitive image-classification models, the translational potential is substantial. In particular, automated label extraction from routine clinical data provides an unprecedented opportunity to efficiently assemble large datasets for local model fine-tuning and rapid adaptation to institutional imaging habits, equipment characteristics, and reporting styles. In our view, this adds an essential tool to the clinical AI toolbox, enabling institutions to calibrate and adapt their AI models for optimal performance once deployed.

Finally, we agree that broadening external validation is essential. Our study included a sizable external cohort from a second academic center, and the close agreement between internal and external performance underscores the robustness of the approach. Expanding to additional institutions, languages, and modalities is a natural next step, particularly for rare or subtle conditions that require larger pooled datasets.

In summary, we appreciate the constructive feedback from Drs. Zhang and Zhang. We hope our clarifications make clear that our study was designed as a proof-of-concept on underrepresented anatomic regions, that the impact of uncertainty was constrained by its low prevalence, that our verification strategy was appropriate for the task, and that we consider broader clinical implementation and multi-institutional expansion essential. Above all, we emphasize the future potential of this approach: by enabling fully automated, high-fidelity conversion of routine radiologic reports into structured datasets, it provides a scalable and practical path toward institution-specific data generation and efficient local model fine-tuning, ultimately supporting more robust, transparent, and clinically adaptable AI in radiology.

Sincerely,

Hanna Kreutzer, MSc, and Sven Nebelung, PhD, MD

(on behalf of the authors)
